# A Phylogenomic and Molecular Markers Based Analysis of the Class *Acidimicrobiia*

**DOI:** 10.3389/fmicb.2018.00987

**Published:** 2018-05-15

**Authors:** Danyu Hu, Guihong Cha, Beile Gao

**Affiliations:** ^1^CAS Key Laboratory of Tropical Marine Bio-resources and Ecology, Guangdong Key Laboratory of Marine Materia Medica, South China Sea Institute of Oceanology, Chinese Academy of Sciences, Guangzhou, China; ^2^University of Chinese Academy of Sciences, Beijing, China

**Keywords:** *Acidimicrobiia*, marine *Acidimicrobiia*, phylogenomics, molecular signatures, conserved signature indels, conserved signature proteins

## Abstract

Recent metagenomic surveys of microbial community suggested that species associated with the class *Acidimicrobiia* are abundant in diverse aquatic environments such as acidic mine water, waste water sludge, freshwater, or marine habitats, but very few species have been cultivated and characterized. The current taxonomic framework of *Acidimicrobiia* is solely based on 16S rRNA sequence analysis of few cultivable representatives, and no molecular, biochemical, or physiological characteristics are known that can distinguish species of this class from the other bacteria. This study reports the phylogenomic analysis for 20 sequenced members of this class and reveals another three major lineages in addition to the two recognized families. Comparative analysis of the sequenced *Acidimicrobiia* species identified 15 conserved signature indels (CSIs) in widely distributed proteins and 26 conserved signature proteins (CSPs) that are either specific to this class as a whole or to its major lineages. This study represents the most comprehensive phylogenetic analysis of the class *Acidimicrobiia* and the identified CSIs and CSPs provide useful molecular markers for the identification and delineation of species belonging to this class or its subgroups.

## Introduction

The class *Acidimicrobiia* is a deep-rooting lineage within the phylum *Actinobacteria*. This class is comprised of few cultivable representatives that were mostly isolated from extremely acidic environments (Zhi et al., 2009; Gao and Gupta, 2012b; Ludwig et al., 2012). Four type species of this class namely, *Acidimicrobium ferrooxidans, Acidithrix ferrooxidans, Ferrimicrobium acidiphilum*, and *Ferrithrix thermotolerans* are extremely acidophilic, with optimal growth pH at around 2.0, and are able to oxidize ferrous iron at relatively fast rates (Cleaver et al., 2007; Ludwig et al., 2012; Norris, 2012). These species were mainly isolated from acidic mine waters or geothermal sites, and were responsible for the regeneration of ferric iron within the acidic ecosystem (Clark and Norris, 1996; Johnson et al., 2009; Jones and Johnson, 2015). In contrast, other members of this class were not acidophiles and inhabited more diverse aquatic environments. For example, neutrophilic *Iamia majanohamensis* was isolated from the abdominal epidermis of a sea cucumber, filamentous “*Candidatus* Microthrix parvicella” (henceforth called M. parvicella) from wastewater sludge, while members of the genus *Ilumatobacter* from estuary sediment or seashore sand (Kurahashi et al., 2009; Matsumoto et al., 2009; Mcllroy et al., 2013).

In spite of few cultivable *Acidimicrobiia* species, metagenomic analyses have revealed that there were many uncultured actinobacterial species belonging to the class *Acidimicrobiia* in freshwater and marine samples (Rheims et al., 1996; Jensen and Lauro, 2008; Ghai et al., 2014). Warnecke et al. (2004) analyzed actinobacterial 16S rRNA genes from freshwater habitats and suggested four most prominent lineages, one of which “acIV lineage” is associated with the order *Acidimicrobiales*. An extensive microbial community composition survey of northwestern Sargasso Sea identified that a marine clade closely related to M. parvicella were abundant in the deep chlorophyll maximum (DCM), with occasional blooms during summer stratification period (Treusch et al., 2009). More recently, Chen et al. (2016) investigated the actinobacterial diversity in the deep sea along the Southwest Indian Ridge and discovered that *Acidimicrobiales* is one of the two most widely distributed and abundant actinobacterial orders in all nine samples from deep sea environments. In addition to the species diversity analyses based on 16S rRNA sequences, further genomic data mining of uncultured *Acidimicrobiia* species suggested that the ecological and metabolic diversity of this class is far underestimated by the culture-dependent species characterization. A metagenomic analysis of Mediterranean DCM assembled four nearly complete genomes for marine *Acidimicrobiales*, and pathway analysis indicated that these species have the capability to assimilate C2 compounds and also derive energy from dimethylsulfoniopropionate, sulfonate, and carbon monoxide (Mizuno et al., 2015). In addition, one of the genomes encodes acidirhodopsin, a novel rhodopsin clade related to freshwater actinorhodopsins (Mizuno et al., 2015).

Although the metagenomic data greatly expanded our knowledge of the species diversity of *Acidimicrobiia*, the current taxonomic frame of this class contains only one order *Acidimicrobiales*, and two families *Acidimicrobiaceae* and *Iamiaceae* with few genera (Ludwig et al., 2012). The taxonomic ranks were determined solely based on 16S rRNA gene sequence analyses and taxon-specific 16S rRNA signature nucleotides using limited representative isolates. To date, despite the availability of seven complete genomes from cultivated *Acidimicrobiia* species and many incomplete genomes from metagenomic data, there is no comprehensive phylogenetic analysis performed to examine the evolutionary relationship within this class. As such, no detailed evolutionary relationship of the uncultured species can be assigned with relation to the known type species of the class *Acidimicrobiia*. (Warnecke et al., 2004; Ghai et al., 2014; Mizuno et al., 2015). In addition, except the branching pattern of these species in phylogenetic trees, no molecular, biochemical or physiological characteristics are known that can clearly distinguish *Acidimicrobiia* species from other *Actinobacteria* (Norris, 2012).

Comparative genomic analyses can lead to discovery of molecular markers that are specific to different higher taxon (e.g., genus level and above), which cannot be easily derived from culture-dependent phenotypic characterization (Gupta and Gao, 2010; Gao and Gupta, 2012a). One important category of these molecular markers is conserved signature indels (CSIs) that are uniquely found in the genes/proteins homologs from a specific group of organisms. Another type of molecular markers are conserved signature proteins (CSPs) that are uniquely shared by a monophyletic group of prokaryotes. The two molecular marker types represent highly reliable characteristics of specific groups of organisms, and they provide novel methods for the identification or delineation of prokaryotic taxonomic units in clear molecular terms (Gao and Gupta, 2012b; Ho et al., 2016; Zhang et al., 2016; Alnajar and Gupta, 2017).

In the present work, a robust phylogenetic tree was constructed for 20 sequenced members of class *Acidimicrobiia* based on 30 universal conserved proteins. The tree clearly showed another three major clusters in addition to the two recognized families within this class, and these clusters may comprise separate families. Besides, comparative analysis of the sequenced *Acidimicrobiia* species identified 15 CSIs in universal proteins and 26 CSPs, which are either specific for this class as a whole or to its major lineages. This study represents the most comprehensive phylogenetic analysis of the class *Acidimicrobiia* and the identified CSIs and CSPs provide useful molecular markers for the identification and demarcation of the members belonging to this class or its subgroups.

## Materials and Methods

### Phylogenetic Analysis

A phylogenetic tree for 20 genome-sequenced members of class *Acidimicrobiia* (Supplementary Table S1) was constructed based on the concatenation of 30 protein sequences, selected from a set of 92 single copy orthologous proteins (Na et al., 2018) and can be retrieved for the most assembled genomes of this class (Supplementary Table S2). Sequences from *Rubrobacter radiotolerans* was used as outgroup to root the tree. Multiple sequence alignments for each protein were performed using the Clustal X 2.1 program (Larkin et al., 2007) and concatenated to produce a single alignment file. The poorly aligned regions of the sequence alignment were removed by the Gblocks 0.91b program (Talavera and Castresana, 2007). The resulting alignment containing 7600 aligned amino acids was used for phylogenetic analysis. A maximum-likelihood (ML) tree was constructed by MEGA 6.0 (Tamura et al., 2013) with the Whelan and Goldman substitution model based on 1000 bootstrap replicates. Another ML tree including more assembled genomes from freshwater *Acidimicrobiia* was constructed based on concatenation of 10 ribosomal protein sequences (Supplementary Table S3). The method applied here was the same as done earlier and the final combined protein alignment used for phylogenetic analysis include 1814 amino acids.

A neighbor-joining (NJ) tree based on sequence alignment of 16S rRNA gene sequences was constructed for the representative strains of cultured *Acidimicrobiia* and some assembled genomes. Full length 16S rRNA sequences were retrieved from Ribosomal Database Project (Cole et al., 2014) or NCBI GenBank, and accession number of each 16S rRNA sequences were summarized in Supplementary Table S4. To root the tree, sequences from three *Rubrobacter* species were used as outgroup. The tree was constructed by MEGA 6.0 using the Kimura 2_parameter model with 1000 bootstrap replicates.

### Identification of CSIs

Conserved signature indels were identified as previously described (Gupta, 2014; Zhang et al., 2016). Briefly, Blastp searches were carried out on all proteins from the genome of *A. ferrooxidans* DSM 10331 (Accession number NC_013124.1) (Clum et al., 2009) against all sequences in the GenBank non-redundant database. Multiple sequence alignments were created for homologs of all available *Acidimicrobiia* species and few other bacteria. These sequence alignments were inspected for any conserved insertions or deletions that were restricted to *Acidimicrobiia* species only and also flanked by at least 5–6 identical or conserved residues in the neighboring 30 ~ 40 amino acids on each side. The indels, whose flanking regions were not conserved, were not further considered and removed. To assess the specificity of the identified indels, detailed BLASTp searches were carried out with a short sequence segment containing the indel and the flanking conserved regions (60–100 amino acids long) against the GenBank database. To further confirm that the identified signatures are restricted to *Acidimicrobiia* homologs, the top 500 BLAST hits with the highest similarity to the query sequence were examined for the presence or absence of these CSIs. Signature files were then created by two programs Sig_Create and Sig_Style (available from Gleans.net) (Gupta, 2014). Due to space limitation, indels containing sequence alignment in all figures and supplementary figures only include those that are found in all *Acidimicrobiia* sequences and few sequences from representative strains of other bacterial groups. It should also be noted that a number of CSIs and CSPs described here are also observed in the assembled genome of endosymbiont cyanobacterium TDX16 deposited by Hebei University of Technology (Accession number NDGV01000834.1, no publication available). We suspect that this unclassified “*Cyanobacteria*” genome assemble is not from DNA of pure culture but rather contamination from multiple bacterial strains since BLASTp searches of individual protein from this assembled genome returns top 5 hits from diverse bacteria, none of which belongs to *Cyanobacteria* and the most frequent best hits are from *Planctomycetes* species. Therefore, sequences from cyanobacterium TDX16 genome is not considered in our analysis.

### Identification of CSPs

BLASTp searches were performed on individual protein from the genome of *A. ferrooxidans* DSM 10331 to identify proteins that are restricted to *Acidimicrobiia* species. These searches were executed against all sequences in the NCBI non-redundant database and the results were then examined manually for proteins with significant hits present only in *Acidimicrobiia* genomes following the same criteria as described in earlier work (Gao et al., 2006; Gao and Gupta, 2012b).

## Results and Discussion

### Phylogenetic Analysis of the Class *Acidimicrobiia* Based on Combined Protein Dataset and 16S rRNA Trees

**FIGURE 1:**
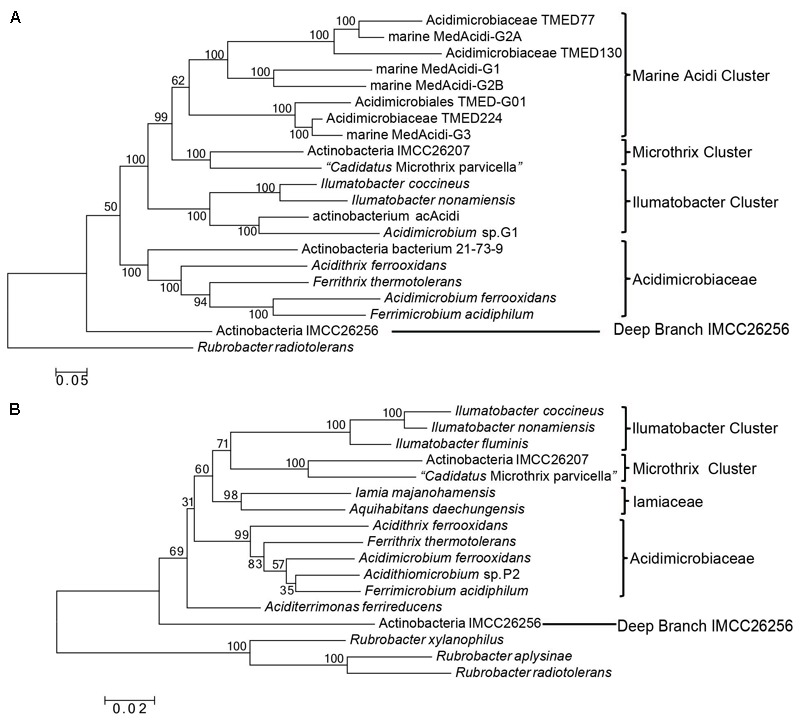
Phylogenetic tree analysis of class *Acidimicrobiia*. **(A)** ML tree for 20 *Acidimicrobiia* species based upon concatenated sequences of 30 conserved proteins. **(B)** Neighbor-joining tree based on full length 16S rRNA gene sequences of all type species within the class *Acidimicrobiia.* Bootstrap values (%) are shown at each node and different clusters that are consistently observed in both phylogenetic trees are marked.

Previous phylogenomic analyses of *Acidimicrobidae* considered two or three fully sequenced species (*A. ferrooxidans, M. parvicella*, and *Ilumatobacter coccineum*) and not more than six assembled genomes from metagenomic sequences (Hugerth et al., 2015; Mizuno et al., 2015). As a result, except confirming the association of these assembled genomes with *Acidimicrobiia*, no detailed evolutionary relationship among these uncultured species and known species can be concluded. In order to get a comprehensive overview of the phylogeny of class *Acidimicrobiia*, a phylogenetic tree was constructed for 7 completely sequenced species of this class and additional 13 assembled genomes from metagenomic data, whose genome information is nearly complete (Supplementary Table S1). The tree was constructed by ML analysis based on concatenation of 30 universally distributed orthologous protein sequences that are mainly involved in translation and transcription (Supplementary Table S2). To date, this tree represents the most comprehensive phylogenetic analysis of the class *Acidimicrobiia* (**Figure 1A**).

In this combined protein tree, an assembled genome “Actinobacteria bacterium IMCC26256” from freshwater sample forms the deepest branch, clearly separated from other *Acidimicrobiia* species. Four type species of the family *Acidimicrobiaceae* together with an assembled genome “Actinobacteria bacterium 21-73-9” from mine drainage metagenome form a well-defined cluster. Based on their branching pattern in the tree and their similar isolation environment, Actinobacteria bacterium 21-73-9 should be affiliated with the family *Acidimicrobiaceae*. The rest in the tree formed three distinctive clusters, and were named after the cultured type species if available found in each cluster namely the “Ilumatobacter Cluster” and “Microthrix Cluster.” A third cluster comprised of assembled genomes from different marine metagenomes is named as “Marine Acidimicrobiia Cluster.”

Since *Iamia*, one of the only two families within this class, do not have any genome sequenced and cannot be used as reference in the combined protein tree analysis, we constructed another phylogenetic tree based on 16S rRNA gene sequences (**Figure 1B**). In this analysis, we try to include all the named species of this class and two assembled genomes from the combined protein tree analysis since the full length 16S rRNA sequences cannot be retrieved from GenBank for the rest of assembled genomes. Consistent with the combined protein tree, Actinobacteria bacterium IMCC26256 formed the deepest branch in the 16S rRNA gene tree of the class *Acidimicrobiia*. In addition, four clusters can be distinguished from each other with high bootstrap scores at each branch node, namely *Acidimicrobiaceae, Iamiaceae*, Microthrix Cluster, and Ilumatobacter Cluster. In both trees shown in **Figure 1**, M. parvicella branched with Actinobacteria bacterium IMCC26207 from freshwater metagenome, distinctive from *Acidimicrobiaceae* and *Iamiaceae*. A recent 16S rRNA analysis of identified *Acidimicrobiia* species and many uncultured environmental clones also indicated that strain IMCC26207 and M. parvicella form a clade clearly separated from *Acidimicrobiaceae* and *Iamiaceae* (Kim et al., 2017). In addition, although the current taxonomic outline placed *Ilumatobacter* within the *Acidimicrobiaceae*, our phylogenetic tree analysis based on both combined protein dataset and 16S rRNA sequences suggest that they are not monophyletic with *Acidimicrobiaceae* species. Hence, in view of the distinctive clustering pattern of Microthrix Cluster and Ilumatobacter Cluster from the two identified families, these two clusters may warrant assignment of novel families within this class. Certainly, this assignment requires additional molecular markers to support the monophyletic relationship of individual cluster.

### Molecular Markers Specific for the Class *Acidimicrobiia*

The availability of complete and nearly complete assembled genomes from class *Acidimicrobiia* provide great resources to explore genomic characteristics that are unique to this class or subgroups within it. CSIs in genes/proteins sequences are important rare genetic changes for understanding bacterial phylogeny (Gao and Gupta, 2007; Gupta and Gao, 2009). The CSIs that serve as useful molecular markers are generally of defined size and their flanking residues are very conserved to ensure their reliability (Gupta, 2014, 2016). Because of the rarity and highly specific nature of such genetic changes, it is less likely that they could arise independently by either convergent or parallel evolution. Most likely, the genetic change responsible for a specific CSI occurred once in a common ancestor of the specific group of species and then passed on vertically to the various descendants. Therefore, CSIs that are restricted to particular clade(s) have generally provided very good phylogenetic markers for evolutionary studies.

**FIGURE 2:**
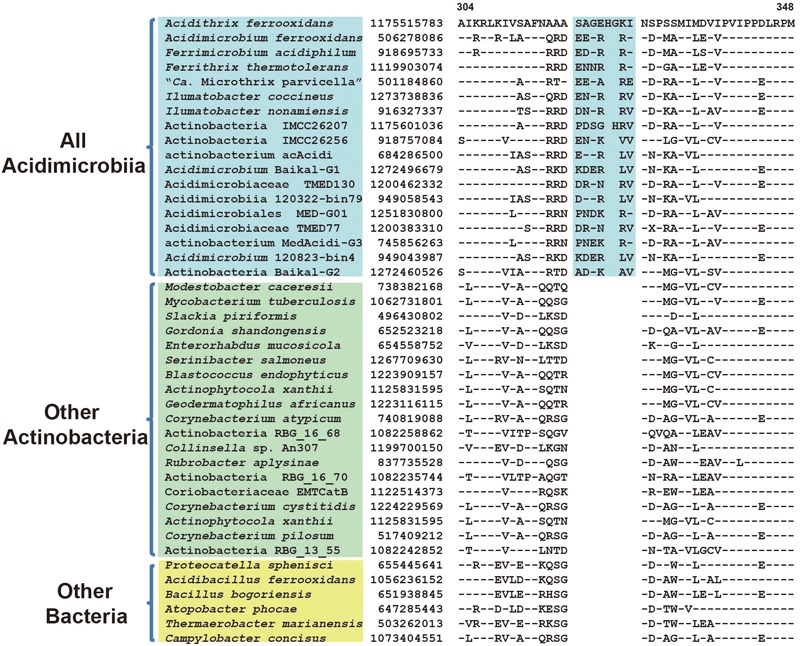
Conserved signature indel (CSI) specific to all *Acidimicrobiia* species. Partial sequence alignment of the protein DNA-directed RNA polymerase subunit beta’ showing a 6 ~ 8aa insertion in a conserved region that is specific for members of the class *Acidimicrobiia*. The dashes in this alignment as well as all other alignments indicate identity with the amino acid on the top line. The GenBank identification numbers of the protein sequences are shown, and the topmost numbers indicate the position of this sequence in the species shown on the top line. Information for other CSIs that are also specific to the class *Acidimicrobiia* are presented in **Table 1** and Supplementary Figures S1,S2.

Comparative analyses of protein sequence alignment from species of class *Acidimicrobiia* and other bacterial groups led to the identification of three CSIs in different conserved proteins that are uniquely shared by all *Acidimicrobiia* species sequenced till date. As shown in **Figure 2**, a 6 ~ 7 amino acids (aa) insertion in a highly conserved region of DNA-directed RNA polymerase subunit beta’ was found to be specific to seven completely sequenced *Acidimicrobiia* species and assembled *Acidimicrobiia* genomes but not present in any other bacteria outside this class. Additionally, a 4 ~ 6 aa insertion in transcription termination factor Rho and a 1 aa deletion in CCA tRNA nucleotidyltransferase were exclusively present in members of class *Acidimicrobiia* (Supplementary Figures S1, S2). For all these CSI containing proteins, homolog sequences of assembled genomes from metagenomic data that belong to class *Acidimicrobiia* were included in the alignment, and all including the deepest branch Actinobacteria bacterium IMCC26256, were found to contain the same CSIs. Thus, these three CSIs constituted distinctive characteristics of the class *Acidimicrobiia* and can be used as molecular markers to define and distinguish species belonging to this class.

In addition to the above CSIs, BLASTp searches of each protein from the genome of type species *A. ferrooxidans* DSM 10331 were carried out to search for CSPs that are unique to class *Acidimicrobiia*. Five proteins were found in almost all sequenced *Acidimicrobiia* genomes including the deepest branch IMCC26256 but not present in any other bacteria outside this class (**Table 2**). Similar to CSIs, these CSPs provide additional molecular markers for class *Acidimicrobiia*. These five proteins identified as hypothetical proteins with unknown function, and due to their specificity, functional studies of these proteins may reveal characteristics that are likely to be unique to members of this class.

**Table 1:** Characteristic of CSIs specific to class *Acidimicrobiia* or its subclades.

Protein name	GI number^a^	Figure number	Indel size	Indel Position^b^	Specificity
DNA-directed RNA polymerase subunit beta’	1175515783	**Figure 2**	6 ~ 8 aa ins^c^	304–348	*Acidimicrobiia*
Transcription termination factor Rho	501180362	Supplementary Figure S1	4 ~ 6 aa ins	539–581	*Acidimicrobiia*
CCA tRNA nucleotidyltransferase	506279427	Supplementary Figure S2	1 aa del^c^	328–369	*Acidimicrobiia*
DNA-directed RNA polymerase subunit beta’	506278086	**Figure 3**	6 ~ 7 aa ins	277–315	*Acidimicrobiia* except IMCC26256
DNA-directed RNA polymerase subunit beta’	506278086	Supplementary Figure S3	7 aa ins	99–139	*Acidimicrobiia* except IMCC26256
Mycothiol S-conjugate amidase	506279235	Supplementary Figure S4	1 aa ins	224–267	*Acidimicrobiia* except IMCC26256
Chlorite dismutase	506279459	Supplementary Figure S5	2 aa ins	117–157	*Acidimicrobiia* except IMCC26256
Polyribonucleotide nucleotidyltransferase	499277639	Supplementary Figure S6	1 aa ins	163–199	*Acidimicrobiia* except IMCC26256
Aspartate-semialdehyde dehydrogenase	502432944	**Figure 4**	3 aa del	200–236	*Acidimicrobiaceae*
Serine hydroxymethyltransferase	506279421	Supplementary Figure S7	6 ~ 8 aa ins	200–236	*Acidimicrobiaceae*
Glutamate decarboxylase	502432855	**Figure 5**	1 aa del	253–289	*Acidimicrobium* and *Ferrimicrobium*
Pyridoxal 5′-phosphate synthase lyase subunit	506278863	Supplementary Figure S8	1 aa ins	183–222	*Acidimicrobium* and *Ferrimicrobium*
Pyridoxal phosphate-dependent aminotransferase	506279158	Supplementary Figure S9	1 aa ins	211–250	*Acidimicrobium* and *Ferrimicrobium*
Type IIA DNA topoisomerase subunit B	916327605	**Figure 6**	2 aa ins	178–210	Ilumatobacter cluster
multifunctional oxoglutarate decarboxylase	521046150	**Figure 7**	6 ~ 7 aa ins	407–451	Microthrix cluster

*^*a*^The GI number represents the GenBank identification number of the protein sequence from one Acidimicrobiia species that contain the specific CSI. ^*b*^The indel region indicates the region of the protein where the described CSI is present. ^*c*^ins, insertion; del, deletion.*

**Table 2:** Conserved signature proteins (CSPs) that are uniquely found in the *Acidimicrobiia* and its subgroups.

Protein product	Specificity	Length	Function
WP_015799038.1	All *Acidimicrobiaceae*	226	Unknown
WP_041661805.1	All *Acidimicrobiaceae*	130	Unknown
WP_015798336.1	All *Acidimicrobiaceae*	99	Unknown
WP_015798164.1	All *Acidimicrobiaceae*	194	Unknown
WP_012226845.1	All *Acidimicrobiaceae*	185	Unknown
WP_015799164.1	*Acidimicrobiaceae*	268	Unknown
WP_015797785.1	*Acidimicrobiaceae*	71	Unknown
WP_041661722.1	*Acidimicrobiaceae*	191	Unknown
WP_041661793.1	*Acidimicrobiaceae*	351	Unknown
WP_015799101.1	*Acidimicrobiaceae*	217	Unknown
WP_015799193.1	*Acidimicrobiaceae*	138	Unknown
WP_015797967.1	*Acidimicrobiaceae*	157	Unknown
WP_015798062.1	*Acidimicrobiaceae*	124	Unknown
WP_041661604.1	*Acidimicrobium,Ferrimicrobium*	418	Unknown
WP_041661730.1	*Acidimicrobium,Ferrimicrobium*	140	Unknown
WP_041661653.1	*Acidimicrobium,Ferrimicrobium*	232	Unknown
WP_015799230.1	*Acidimicrobium,Ferrimicrobium*	139	Unknown
WP_015799176.1	*Acidimicrobium,Ferrimicrobium*	177	Unknown
WP_015799100.1	*Acidimicrobium,Ferrimicrobium*	255	Unknown
WP_015799084.1	*Acidimicrobium,Ferrimicrobium*	374	Unknown
WP_015798540.1	*Acidimicrobium,Ferrimicrobium*	145	Unknown
WP_015798531.1	*Acidimicrobium,Ferrimicrobium*	63	Unknown
WP_015798470.1	*Acidimicrobium,Ferrimicrobium*	743	Unknown
WP_015798187.1	*Acidimicrobium,Ferrimicrobium*	166	Unknown
WP_015797784.1	*Acidimicrobium,Ferrimicrobium*	200	Unknown
WP_015798639.1	*Acidimicrobium,Ferrimicrobium*	261	Unknown

### Molecular Signatures for Some of the Subclades of *Acidimicrobiia*

**FIGURE 3:**
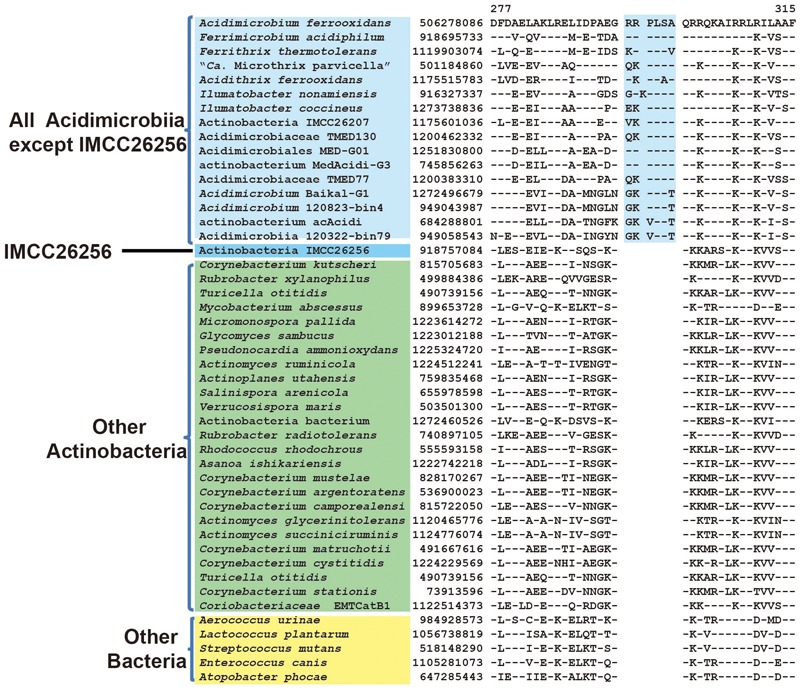
Conserved signature indel specific to all *Acidimicrobiia* species except strain IMCC26256. Partial sequence alignment of DNA-directed RNA polymerase subunit beta’ showing a 6 ~ 7 aa insertion that is specific for all *Acidimicrobiia* except Actinobacteria bacterium IMCC26256. Information for other CSIs specific for this clade are presented in **Table 1** and Supplementary Figures S3–S6.

As mentioned earlier, uncultivated Actinobacteria bacterium IMCC26256 formed the deepest branch in both phylogenetic trees based on combined protein dataset and 16S rRNA, which suggest that this species might be the earliest branch within known *Acidimicrobiia* species to date. In our analysis, we have identified five CSIs in four different widely distributed conserved proteins that are uniquely shared by all members of class *Acidimicrobiia* except strain IMCC26256. Although missing in IMCC26256 genome, these CSIs are not found in any other non-*Acidimicrobiia* species. One example of these CSIs is shown in **Figure 3**. In a highly conserved region of DNA-directed RNA polymerase subunit beta’, a 7 aa insert is unique to all *Acidimicrobiia* species but missing in IMCC26256 genome. Additional four CSIs showing similar specificity are presented in Supplementary Figures S3–S6. The absence of the identified CSIs in homologs of IMCC26256 genome are not due to lateral gene transfer since the best BLASTp hit of these CSIs containing proteins in IMCC26256 genome are homologous sequences of *Acidimicrobiia* species rather than other bacterial groups. There are two possible explanations for the presence of these five CSIs. First, these CSIs evolved in a common ancestor of all *Acidimicrobiia* but subsequently lost in IMCC26256 genome; second, these CSIs were introduced in the common ancestor of other *Acidimicrobiia* lineages after the branch of Actinobacteria bacterium IMCC26256. Although we cannot discriminate which of the two evolutionary scenarios account for the absence of these five CSIs in IMCC26256 genome, the unique presence of these CSIs in the rest of *Acidimicrobiia* species indicated that they constitute distinctive characteristics of this class.

**FIGURE 4:**
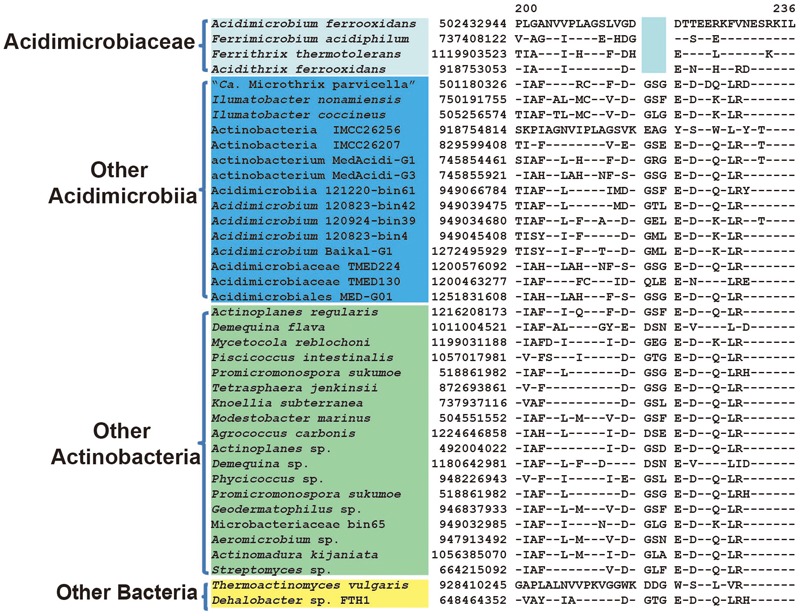
Conserved signature indel specific to the family *Acidimicrobiaceae*. Partial alignment of the protein aspartate-semialdehyde dehydrogenase showing a 3 aa deletion that is specific for the family *Acidimicrobiaceae.* Information for other CSIs that are specific for the family *Acidimicrobiaceae* are presented in **Table 1** and Supplementary Figure S7.

Our analysis also identified two CSIs that are specifically shared by members of *Acidimicrobiaceae*, namely *A. ferrooxidans, F. acidiphilum, F. thermotolerans*, and *Acidithrix ferrooxidans*. These CSIs include a 3 aa deletion in aspartate-semialdehyde dehydrogenase (**Figure 4**) and a 6 ~ 8 aa insertion in serine hydroxymethyltransferase (Supplementary Figure S7). They are exclusively present in the above four species belonging to the family *Acidimicrobiaceae* but not found in any other species. In addition, we also identified eight CSPs that are unique to these four species (**Table 2**). In contrast, no CSIs or CSPs were found that are uniquely shared by these species and *Ilumatobacter* species, which are currently assigned under the family *Acidimicrobiaceae*. These results suggest that most likely *Ilumatobacter* and the above four species are not monophyletic, consistent with the results from phylogenetic tree analysis. Therefore, *Ilumatobacter* should not be placed under the family *Acidimicrobiaceae*. Moreover, these two CSIs and eight CSPs provide distinctive molecular markers for the family *Acidimicrobiaceae* that can be used to define and delineate species belonging to this family.

**FIGURE 5:**
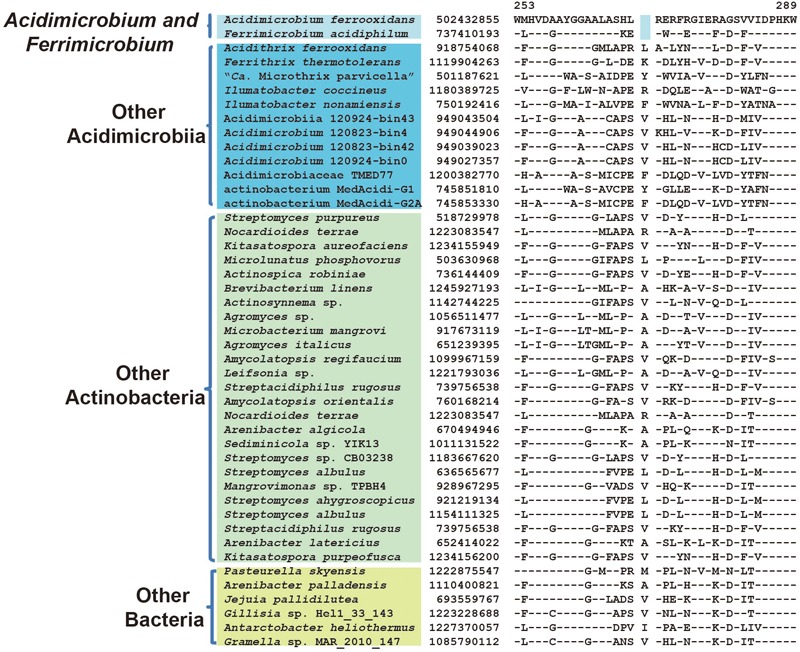
Conserved signature indel specific to genera *Acidimicrobium* and *Ferrimicrobium.* A 1 aa deletion in the protein glutamate decarboxylase that is uniquely shared by *A. ferrooxidans* and *F. acidiphilum.* Information of other CSIs specific for this cluster is present in **Table 1**, and Supplementary Figures S8, S9.

Among members of the family *Acidimicrobiaceae, A. ferrooxidans*, and *F. acidiphilum* formed a clade in phylogenetic trees, thereby indicating a more closer relationship among the two than from the other species of this family (**Figure 1**). Supporting this relationship, we have identified three CSIs in different proteins that were present only in *A. ferrooxidans* and *F. acidiphilum*. These CSIs include: 1 aa deletion in glutamate decarboxylase (**Figure 5**), a 1 aa insertion in pyridoxal 5′-phosphate synthase lyase subunit PdxS, and a 1 aa insertion in pyridoxal phosphate-dependent aminotransferase (Supplementary Figures S8, S9). Besides, 13 CSPs were identified as unique proteins shared by both *A. ferrooxidans* and *F. acidiphilum* (**Table 2**).

**FIGURE 6:**
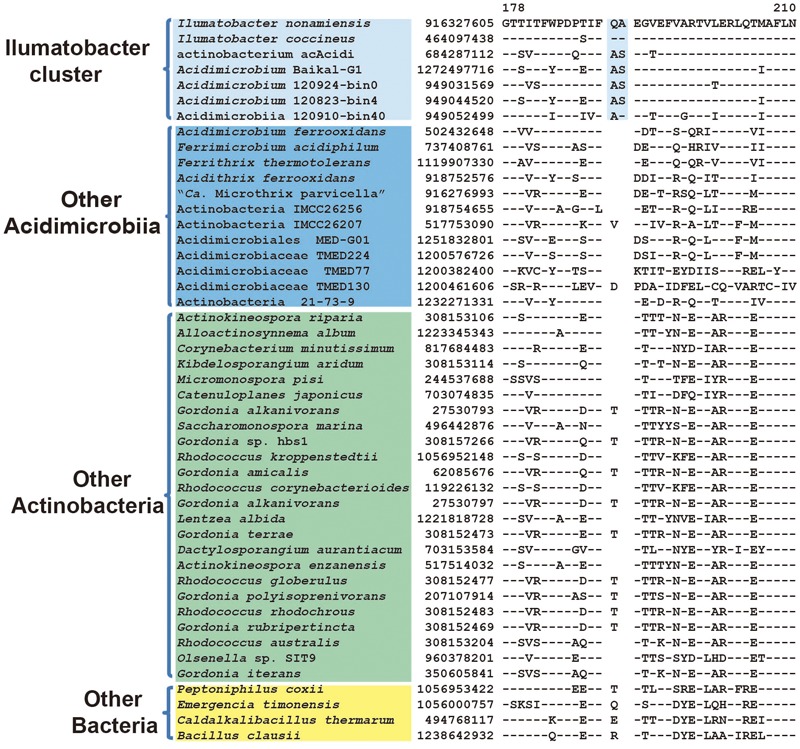
Conserved signature indel specific to Ilumatobacter cluster. A 2 aa CSI in the protein type IIA DNA topoisomerase subunit B that is specific for the Ilumatobacter cluster.

As revealed by phylogenetic tree analysis, *Ilumatobacter* species branched together with assembled genome “actinobacterium acAcidi” from freshwater and “Acidimicrobium sp. Baikal-G1” from lake water. Two other phylogenetic trees based on 16S rRNA and combined protein dataset with limited *Acidimicrobiia* species also indicate that *Ilumatobacter* formed a cluster with acIV freshwater lineage (Hugerth et al., 2015; Mizuno et al., 2015). In our CSIs searches, we identified one CSI, a 2 aa insertion in type IIA DNA topoisomerase subunit B, that are uniquely shared by *Ilumatobacter* species and multiple assembled *Acidimicrobiia* genomes from freshwater samples (**Figure 6**). To further confirm the relationship of numbers of the genus *Ilumatobacter* and additional assembled freshwater *Acidimicrobiia* genomes, we constructed another phylogenetic tree based on ten ribosomal proteins for which sequences can be retrieved from the incomplete genomes of freshwater species (Supplementary Figure S10). Indeed, *Ilumatobacter* species formed a well-defined cluster with freshwater *Acidimicrobiia* species supported by high bootstrap score at the branch node. Taken together, the identified CSI and phylogenetic tree analysis suggested that members of the genus *Ilumatobacter* genus showed more close relationship with freshwater *Acidimicrobiia* lineage, and they should be assigned as an independent family different from that of the parent family *Acidimicrobiaceae.*

**FIGURE 7:**
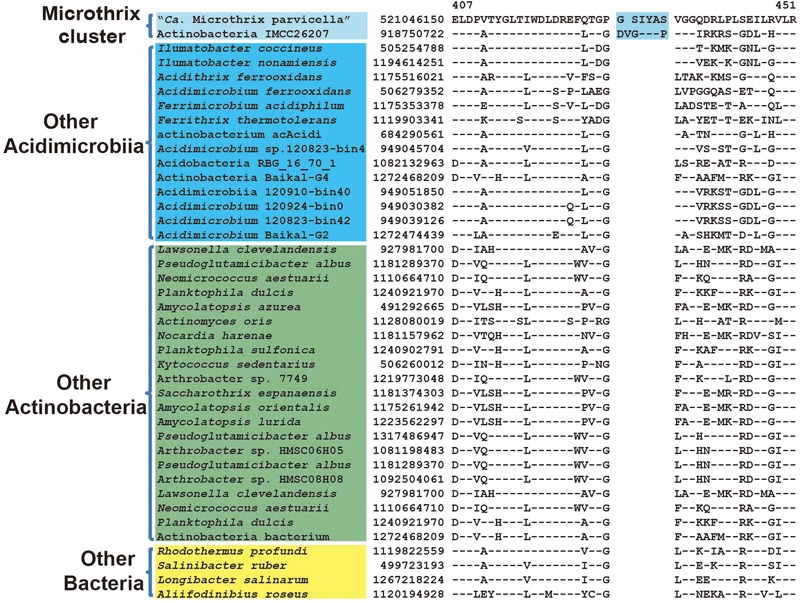
Conserved signature indel specific to Microthrix cluster. Partial sequence alignment of multifunctional oxoglutarate decarboxylase showing a 6 ~ 7aa insertion that is specific for the Microthrix cluster comprising of M. parvicella and strain IMCC26207.

Microthrix parvicella was frequently retrieved from activated sludge wastewater treatment plants and had characteristic long unbranched filamentous morphology (Rossetti et al., 2005). Previous phylogenetic analyses indicated that M. parvicella belonged to *Acidimicrobiia* but formed a separate branch from the other type species of this class (Mizuno et al., 2015). In our phylogenetic trees based on both 16S rRNA and combined protein dataset (**Figure 1**), a recently published assembled genome of freshwater isolate, strain IMCC26207 with proposed species name “*Candidatus* Limnosphaera aquatica” (Kim et al., 2017), formed a distinctive clade with M. parvicella supported by high bootstrap score. This is the most closely related genome for M. parvicella reported to date (Kim et al., 2017). A 6 ~ 7 aa insertion in a highly conserved region of multifunctional oxoglutarate decarboxylase was identified to be specific to M. parvicella and IMCC26207 (**Figure 7**), which provide a potential molecular marker for Microthrix cluster but awaits confirmation with more homologous sequences from closely related species.

## Conclusion

In spite of the abundance of *Acidimicrobiia* in diverse aquatic habitats and their important role in biogeochemical cycling, presently there is limited study on the phylogeny of this deep branch class of the phylum *Actinobacteria*. The current taxonomic framework based on few cultivated species need to be updated to serve as guide map for increasing metagenomic investigation of species diversity of this class. In the present work, we have performed detailed phylogenomic analysis of sequenced *Acidimicrobiia* species and assembled genomes, which revealed three distinctive clusters namely Ilumatobacter cluster, Microthrix Cluster and Marine Acidic Cluster, in addition to the only two recognized families. More importantly, we have identified multiple CSIs in different proteins and CSPs that are specific to either class *Acidimicrobiia* or certain lineages within it. These genomic signature sequences can be used as molecular markers to define or delineate class *Acidimicrobiia* or its subgroups at higher taxonomic ranks, in addition to the current standard based on 16S rRNA alone. The class *Acidimicrobiia* currently only consists of two families-*Acidimicrobiaceae* and *Iamiaceae*, the latter of which has no genomes sequenced, and thus no CSIs/CSPs can be identified. In total, we have discovered two CSIs and eight CSPs specific for four species of the family *Acidimicrobiaceae*, but not present in species of the genera *Microthrix* and *Ilumatobacter* genera. Based on the clustering pattern of phylogenetic trees presented in **Figure 1** and the identified CSIs for Ilumatobacter Cluster and Microthrix Cluster, these two clades are not monophyletic with type species of *Acidimicrobiaceae* and should be defined as independent families. Furthermore, according to our phylogenomic analysis, *Acidimicrobiia* species from marine environments formed a cluster distinct from the other cultured type species, suggesting that these marine *Acidimicrobiia* might share unique genotypic and phenotypic characteristics. Hence, it is of much interest to identify molecular markers that are uniquely shared by marine *Acidimicrobiia* in the future.

Finally, both CSI-containing proteins and CSPs perform different functions in the bacterial cells, although the function of most of these molecular markers are unknown at present. Due to their specificity, the function of these CSIs and CSPs might be some characteristics unique to the specific taxon that contain them. For example, one *Actinobacteria*-specific CSP, ParJ (SCO1662), was functionally characterized as regulating the polymerization of ParA protein and affecting chromosome segregation and cell division during *Streptomyces* sporulation (Ditkowski et al., 2010). The *Acidimicrobiia*-specific CSIs and CSPs presented here provide novel targets for functional studies, which may reveal yet undiscovered features that are unique to species of this diverse class.

## Author Contributions

DH carried out comparative analyses of the *Acidimicrobiia* genomes to identify signatures reported here, DH and GC constructed the phylogenetic trees. BG and DH were responsible for the writing and editing of the manuscript. All of the work was carried out under the direction of BG.

## Conflict of Interest Statement

The authors declare that the research was conducted in the absence of any commercial or financial relationships that could be construed as a potential conflict of interest. The reviewer SN and handling Editor declared their shared affiliation.
