# Supplementary material for: A Phylogenomic and Molecular Markers Based Analysis of the Class Acidimicrobiia
Source: Front Microbiol. 2018 May 15;9:987. doi: 10.3389/fmicb.2018.00987 (PMC5962788; doi:10.3389/fmicb.2018.00987)
Supplement: Supplementary file 1 [file Data_Sheet_1.PDF]

Supplementary Material

A phylogenomic and molecular markers based analysis of the class *Acidimicrobiia*

Danyu Hu, Guihong Cha and Beile Gao*

*** Correspondence:** gaob@scsio.ac.cn

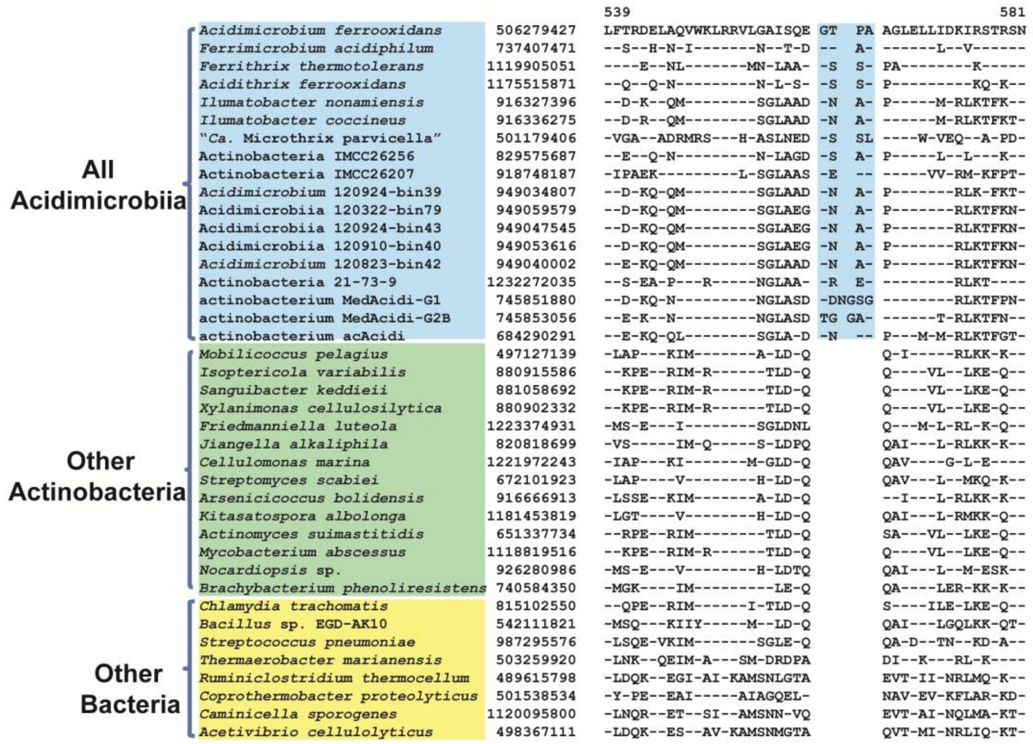

Supplementary Figure 1. Partial sequence alignment of transcription termination factor Rho showing a 4~6 aa insertion that is specific to the class *Acidimicrobiia*.

.

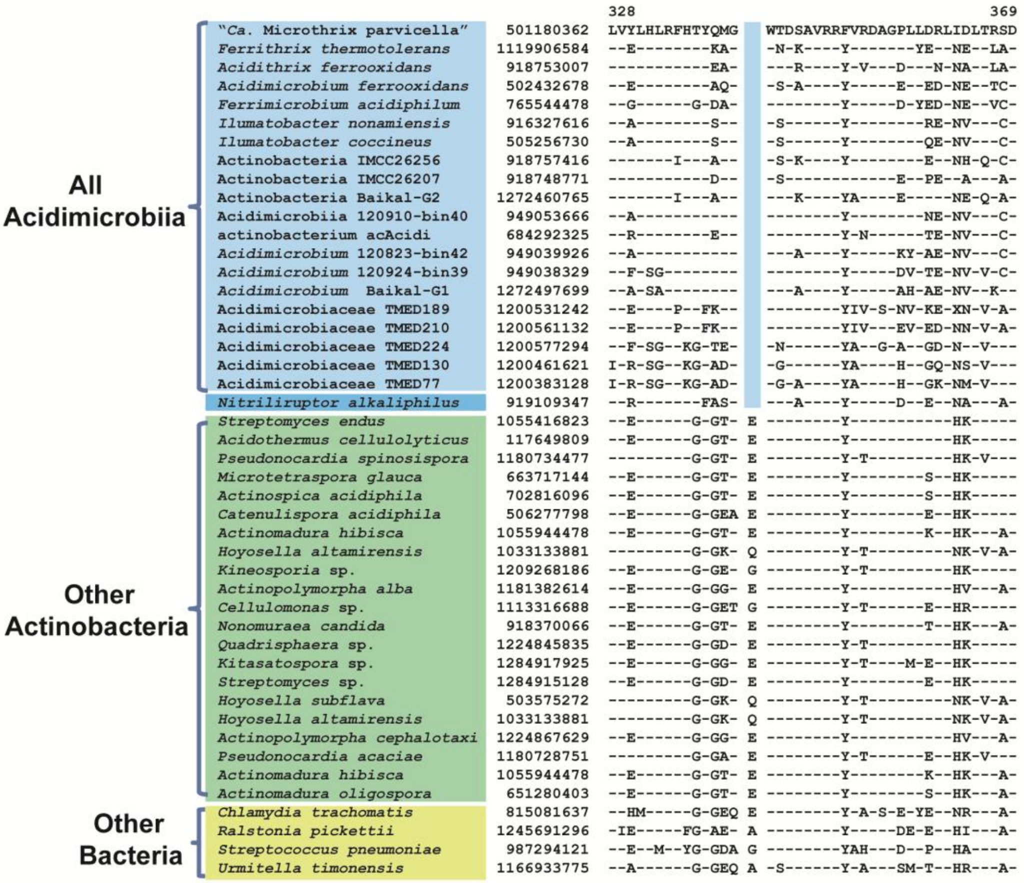

Supplementary Figure 2. Partial sequence alignment of CCA tRNA nucleotidyltransferase showing a 1 aa deletion that is specific to the class *Acidimicrobiia*. Besides Acidimicrobiia, a similar deletion is also present in species *Nitriliruptor alkaliphilus*.

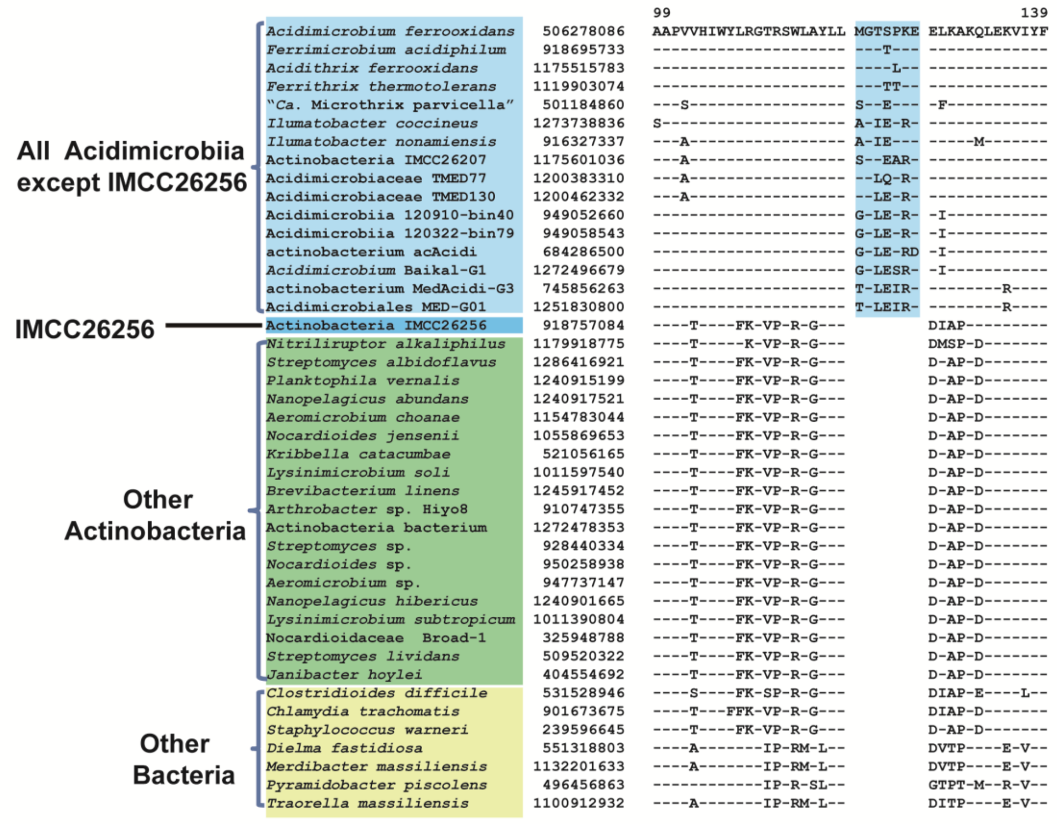

Supplementary Figure 3. Partial sequence alignment of DNA-directed RNA polymerase subunit beta' showing a 7 aa insertion that is specific to the class *Acidimicrobiia* except Actinobacteria bacterium IMCC26256.

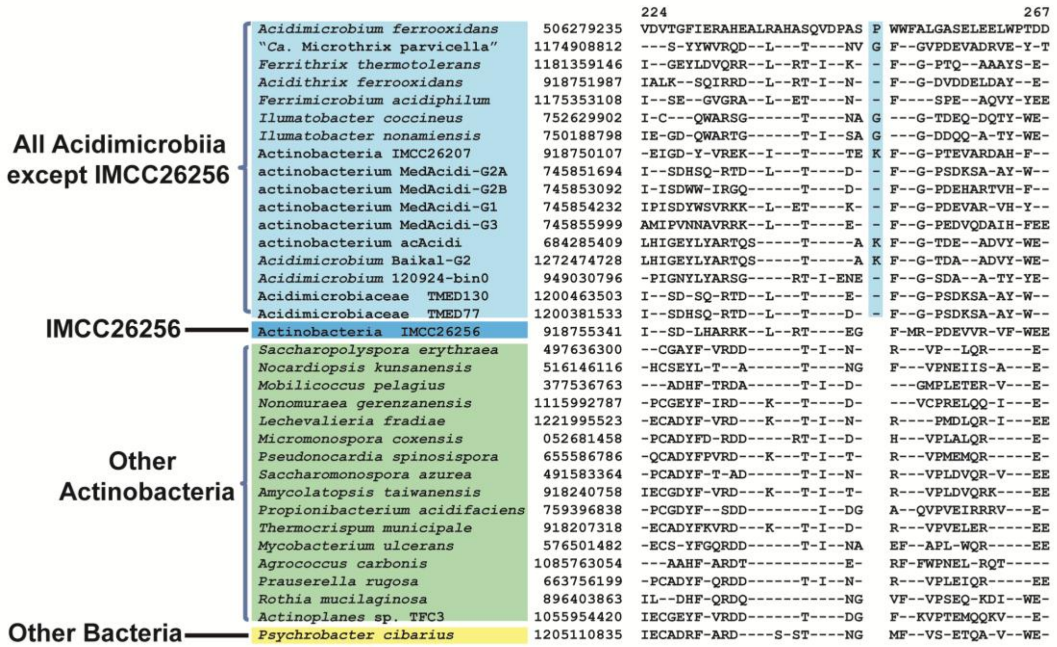

Supplementary Figure 4. Partial sequence alignment of mycothiol S-conjugate amidase showing a 1 aa insertion that is specific to the class *Acidimicrobiia* except Actinobacteria bacterium IMCC26256.

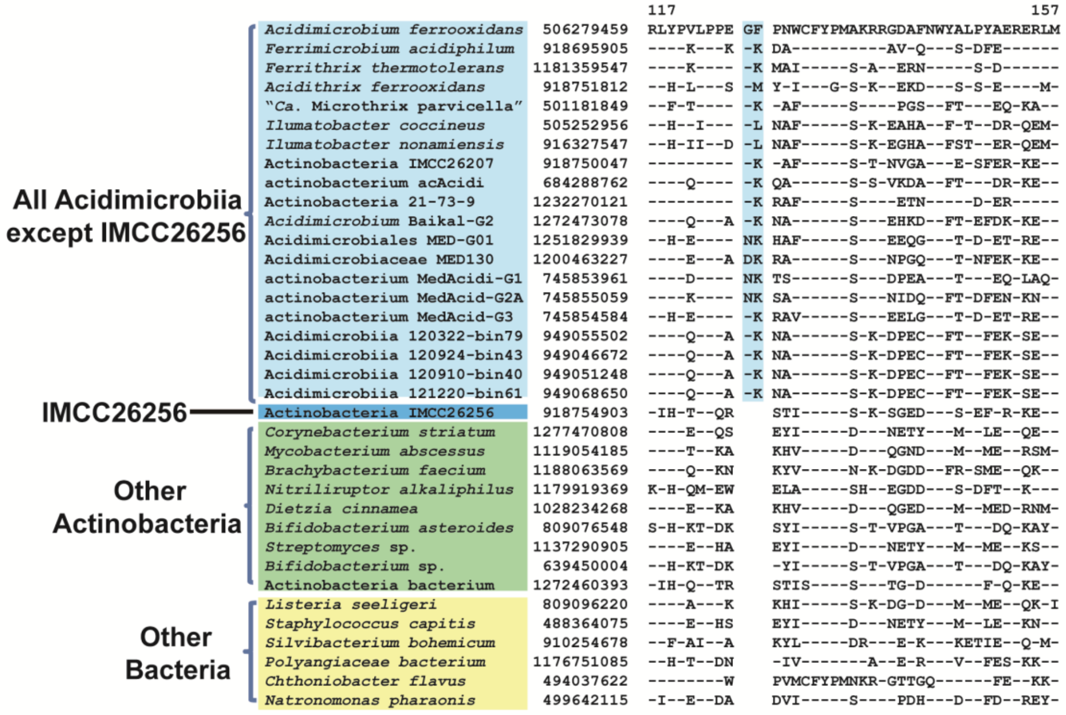

Supplementary Figure 5. Partial sequence alignment of chlorite dismutase showing a 2 aa insertion that is specific to the class *Acidimicrobiia* except the species Actinobacteria bacterium IMCC26256.

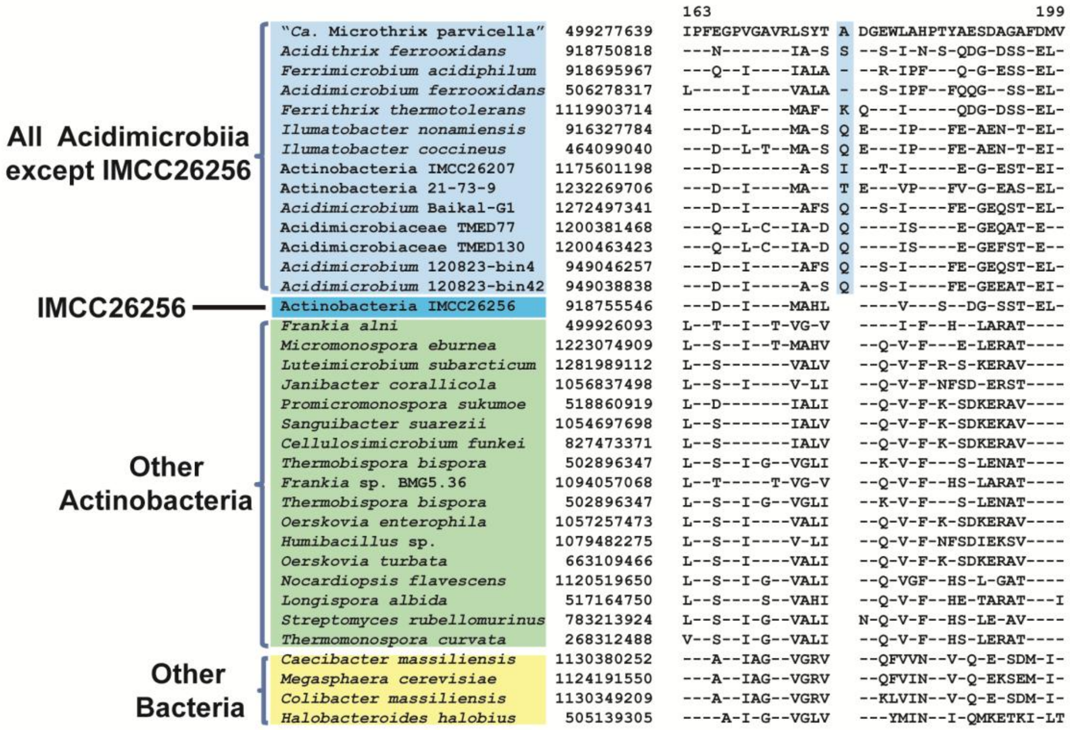

Supplementary Figure 6. Partial sequence alignment of polyribonucleotide nucleotidyltransferase showing a 1 aa insertion that is specific to the class *Acidimicrobiia* except Actinobacteria bacterium IMCC26256.

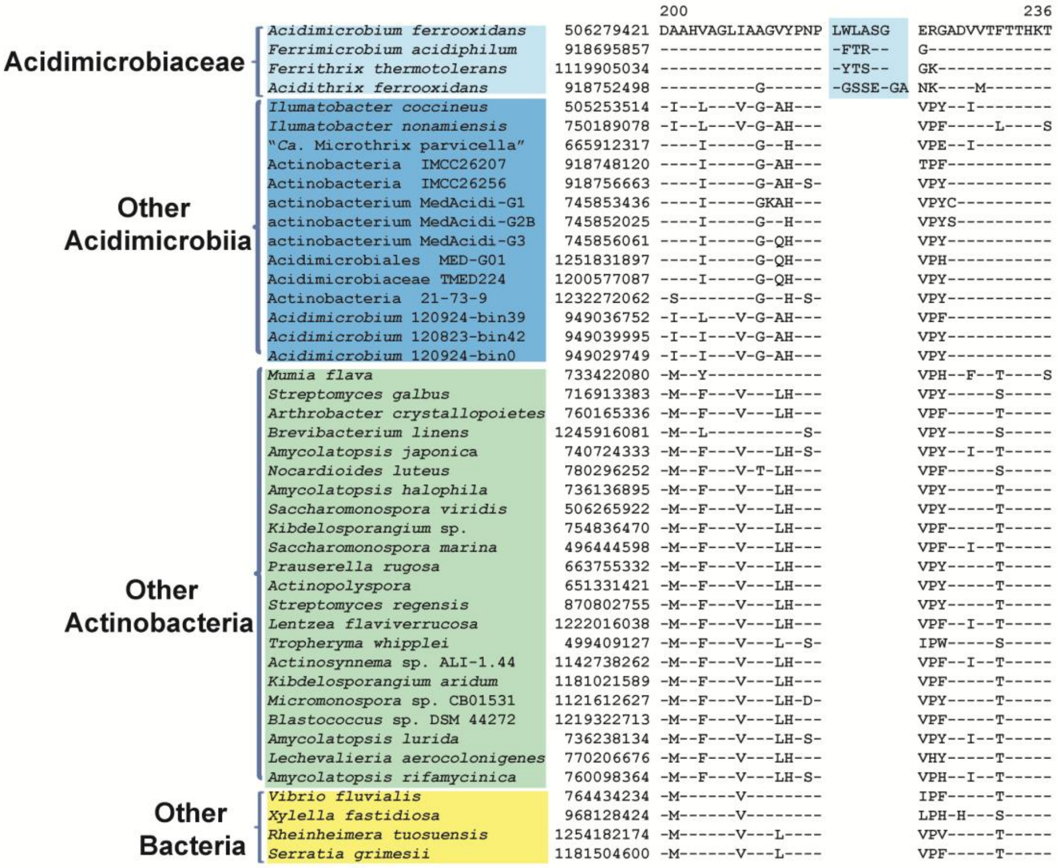

Supplementary Figure 7. Partial sequence alignment for serine hydroxymethyltransferase protein showing a 6~8 aa insertion that is specific to the family *Acidimicrobiaceae*.

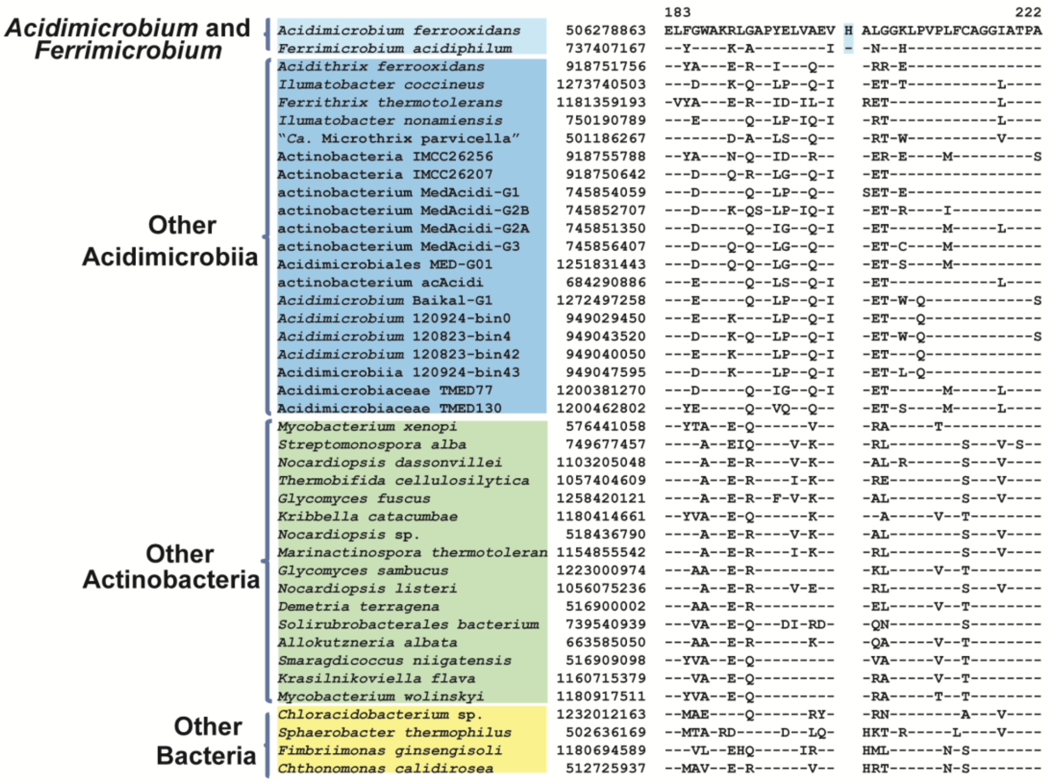

Supplementary Figure 8. Conserved signature indel in the proteins pyridoxal 5'-phosphate synthase lyase subunit PdxS, which is specific to the genera *Acidimicrobium* and *Ferrimicrobium*.

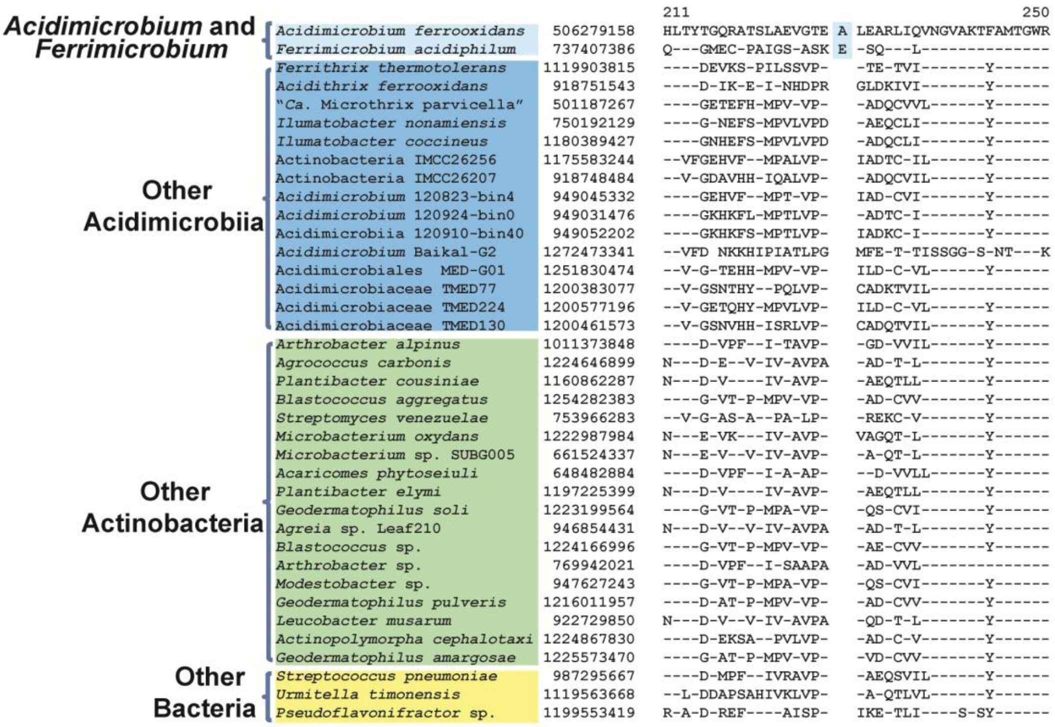

Supplementary Figure 9. Conserved signature indel in the protein pyridoxal phosphate-dependent aminotransferase that is specific to the genera *Acidimicrobium* and *Ferrimicrobium*.

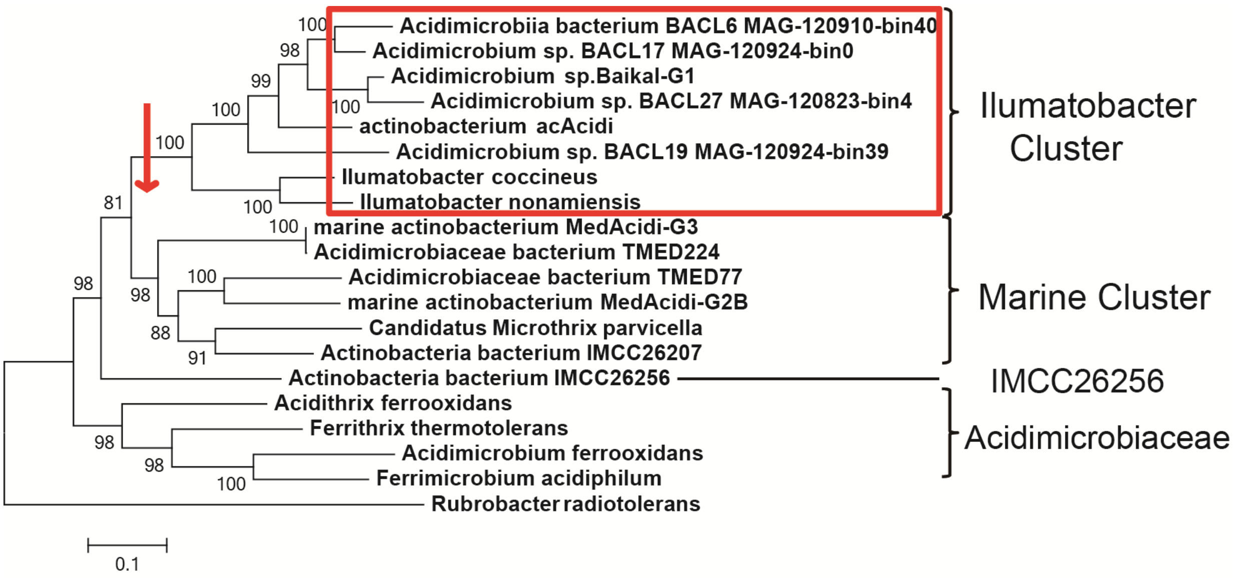

Supplementary Figure 10. A Maximum-likelihood tree based on concatenated sequences of ten ribosomal proteins from 19 genome-sequenced members of the class *Acidimicrobiia*. The Ilumatobacter cluster including two sequenced *Ilumatobacter* species and multiple assembled genomes from freshwater metagenome was highlighted by red box, and the branch node where the CSI presented in Figure 6 was likely introduced was labeled by red arrow.

Supplementary Table 1
Genomic characteristic of the species within the class *Acidimicrobiia*

Strain	Length (Mb)	Protein count	GC%	Accession No.	Reference [PMID]
Acidithrix ferrooxidans Py-F3	4.02	3395	47.7	GCA_000949295.1	25931603
Ferrithrix thermotolerans DSM 19514	2.49	2238	51.1	GCA_900128965.1	19406797
Acidimicrobium ferrooxidans DSM 10331	2.16	2034	68.3	GCA_000023265.1	21304635
Ferrimicrobium acidiphilum DSM 19497	3.01	2684	55.3	GCA_000745905.1	19406797
“Candidatus Microthrix parvicella” RN1	3.69	3774	66.55	GCA_000455525.1	23144412
Ilumatobacter nonamiensis YM16-303	4.28	3862	67	GCA_000350145.1	23524358
Ilumatobacter coccineus YM16-304	4.83	4203	67.3	GCA_000348785.1	23524358
Actinobacteria bacterium IMCC26207	3.32	2,975	57.3	GCA_001025035.1	28173694
Actinobacteria bacterium IMCC26256	2.46	2,275	55.4	GCA_001023575.1	n.a
Actinobacteria bacterium 21-73-9	1.96	1,987	72.5	GCA_002255565.1	n.a
marine actinobacterium MedAcidi-G1	1.68	1361	42.4	GCA_000817085.1	25670777
marine actinobacterium MedAcidi-G2A	1.37	1146	43.1	GCA_000817095.1	25670777
marine actinobacterium MedAcidi-G2B	1.44	1149	44.8	GCA_000817115.1	25670777
marine actinobacterium MedAcidi-G3	2.11	1678	51.4	GCA_000817105.1	25670777
actinobacterium acAcidi	2.69	2884	52.2	GCA_000754585.1	25355242
Acidimicrobium sp.Baikal G1	1.45	1346	50.3	GCA_002737635.1	26667648
Acidimicrobiales bacterium MED-G01	1.96	1941	53	GCA_002457435.1	n.a
Acidimicrobiaceae bacterium TMED224	1.44	1404	51.1	GCA_002169095.1	n.a
Acidimicrobiaceae bacterium TMED77	1.39	1357	42.9	GCA_002171605.1	n.a
Acidimicrobiaceae bacterium TMED130	1.46	1432	44.2	GCA_002170115.1	n.a

Supplementary Table 2
Details of the proteins used in phylogenetic analysis presented in Figure 1A

Protein name	Length(aa)#	COG ID
Dimethyladenosine transferase (KsgA)	279	COG0030
30S ribosomal protein S12	182	COG0048
30S ribosomal protein S7	156	COG0049
Elongation factor Tu	395	COG0050
3-isopropylmalate dehydratase, small subunit	202	COG0066
50S ribosomal protein L3	213	COG0087
50S ribosomal protein L4	214	COG0088
50S ribosomal protein L23	98	COG0089
50S ribosomal protein L2	278	COG0090
ribosomal protein L22	218	COG0091
30S ribosomal protein S3	288	COG0092
50S ribosomal protein L14	122	COG0093
30S ribosomal protein S5	183	COG0098
30S ribosomal protein S13	125	COG0099
30S ribosomal protein S19	93	COG0185
30S ribosomal protein S17	90	COG0186
DNA gyrase, B subunit	647	COG0187
peptidyl-tRNA hydrolase	192	COG0193
50S ribosomal protein L16	145	COG0197
50S ribosomal protein L24	101	COG0198
50S ribosomal protein L15	166	COG0200
Preprotein translocase subunit SecY	431	COG0201
UvrD/REP helicase	715	COG0210
Peptide chain release factor 1	357	COG0216
ribosomal protein L22	78	COG0255
30S ribosomal protein S20	89	COG0268
elongation factor G	695	COG0480
Elongation factor 4	595	COG0481

tRNA N6-adenosine threonylcarbamoyltransferase	347	COG0533
UvrABC system protein B	667	COG0556

Note: The phylogenetic tree for the class *Acidimicrobiia* presented in Figure 1(A) was constructed based upon concatenated sequence alignment of the above proteins. # Protein length is from *Acidimicrobium ferrooxidans* DSM10331.

Supplementary Table 3.
Details of the proteins used in phylogenetic analysis presented in Supplementary Figure S10

Protein	Length(aa#)	COG ID
50S ribosomal protein L2	278	COG0090
50S ribosomal protein L3	213	COG0087
50S ribosomal protein L4	214	COG0088
50S ribosomal protein L5	189	COG0094
50S ribosomal protein L6	179	COG0097
50S ribosomal protein L13	154	COG0102
30S ribosomal protein S3	288	COG0092
30S ribosomal protein S5	183	COG0098
30S ribosomal protein S9	130	COG0103
30S ribosomal protein S13	125	COG0099

Note: List of proteins selected for phylogenetic analysis including more assembled freshwater *Acidimicrobiia* genomes presented in Supplementary Figure S10.

Supplementary Table 4
Summary of 16S rRNA sequences used in phylogenetic tree construction in Figure 1B

Strain	GI No.	length(bp)
Acidithrix ferrooxidans Py-F3	441084445	1192
Ferrithrix thermotolerans Y005	343202455	1331
Acidimicrobium ferrooxidans DSM 10331	444303967	1498
Ferrimicrobium acidiphilum T23	343198427	1466
“Candidatus Microthrix parvicella” RN1	223016922	1416
Ilumatobacter nonamiensis YM16-303	631251516	1443
Ilumatobacter coccineus YM16-304	631251517	1443
Actinobacteria bacterium IMCC26207	829597323	1526
Actinobacteria bacterium IMCC26256	829574018	1523
Acidithiomicrobium sp. P2	GQ225721	1488
Aciditerrimonas ferrireducens IC-180	631251774	1483
Rubrobacter aplysinae RV113	959494996	1512
Rubrobacter radiotolerans P 1	602270569	1532
Rubrobacter xylanophilus PRD-1	645322440	1509
Iamia majanohamensis NBRC 102561	343200947	1474
Aquihabitans daechungensis CH22-21	848212582	1439
Ilumatobacter fluminis YM22-133	343200946	1439

The GI number represents the GenBank Identification number of 16S rRNA sequences.
